# A New Hammer to Crack an Old Nut: Interspecific Competitive Resource Capture by Plants Is Regulated by Nutrient Supply, Not Climate

**DOI:** 10.1371/journal.pone.0029413

**Published:** 2012-01-11

**Authors:** Clare J. Trinder, Rob W. Brooker, Hazel Davidson, David Robinson

**Affiliations:** 1 Institute of Biological and Environmental Sciences, University of Aberdeen, Aberdeen, United Kingdom; 2 The James Hutton Institute, Aberdeen, United Kingdom; University of New South Wales, Australia

## Abstract

Although rarely acknowledged, our understanding of how competition is modulated by environmental drivers is severely hampered by our dependence on indirect measurements of outcomes, rather than the process of competition. To overcome this, we made direct measurements of plant competition for soil nitrogen (N). Using isotope pool-dilution, we examined the interactive effects of soil resource limitation and climatic severity between two common grassland species. Pool-dilution estimates the uptake of total N over a defined time period, rather than simply the uptake of ^15^N label, as used in most other tracer experiments. Competitive uptake of N was determined by its available form (NO_3_^−^ or NH_4_^+^). Soil N availability had a greater effect than the climatic conditions (location) under which plants grew. The results did not entirely support either of the main current theories relating the role of competition to environmental conditions. We found no evidence for Tilman's theory that competition for soil nutrients is stronger at low, compared with high nutrient levels and partial support for Grime's theory that competition for soil nutrients is greater under potentially more productive conditions. These results provide novel insights by demonstrating the dynamic nature of plant resource competition.

## Introduction

The concept of competition among individuals is central to ecological theory. It is often considered a determinant of the demographic success of individuals and populations [1], the genotypic composition of communities and hence biodiversity [2], [3] and the evolution of phenotypic strategies and traits [4]. Yet much uncertainty persists about the role of competition in regulating populations, structuring communities, and driving evolution. The unresolved questions surrounding competition are far from being trivial; they touch on many key theoretical and applied issues. For example, models to predict organismal responses to environmental change have struggled to incorporate the influence of biotic interactions, of which competition is an obvious component [5], [6]. This is, in part, because there is enduring and ongoing debate about the circumstances under which such interactions play a substantial role in regulating organismal success and hence community composition [7]–[10]. However, despite almost universal acceptance of its potential ecological importance, and consequently the enormous attention devoted to it, competition is notoriously resistant to direct and unambiguous measurement [11], [12].

Many supposed measures of plant competition have been used [13] including, for example, biomass production by neighbouring individuals [14]–[16] or, less often, changes in size of populations occupying the same habitat [17], [18]. Yet all of these measure an *outcome* of, rather than the *process* of, competition, i.e., they are proxies for competition. Competition *sensu stricto* – the contest for an essential resource by neighbouring individuals that are exploiting the same finite supply [4] – is seldom measured directly, *in situ*, or in real time in terms of the simultaneous fluxes of resources into competing individuals [19]. Of course, there are many possible definitions of competition, which does not aid clarity, but the explicit definition that we use here matches the general theoretical recognition of the concept, if not its practical application [20]. The practical and theoretical distinctions between direct and indirect measurements of competition are rarely appreciated. The relative ease and practicability of using proxy indicators of competition is understandable. But at the same time this can compromise the interpretation of competition experiments and hinder the development of ecological theory which is, more commonly, based on underlying mechanisms of interactions between individuals. To quote Williams [21], “The basic problem … is the very common one of the easily measured variables not being the theoretically important ones.”

Opacity on this subject has been reinforced to some extent by the widespread use of ‘competition indices’ to estimate the effects of competition on individuals [22]. These indices, formed by combining several primary response variables such as the biomass of a competitor relative to that of an isolated control, are used routinely, but their analysis can be statistically problematic making their interpretation potentially ambiguous [23], [24].

As an alternative to these indirect approaches, a few studies have attempted to measure the competitive contest for resources directly, but all of these have limitations. For example, ^32^P introduced into soil containing the root systems of presumed competitors has been used to measure: competitive interactions in relation to root penetration at different depths [25]; competition between a grass and a desert shrub [26]; the effects of defoliation [19]; and responses by plants to patchy soil resources [27]. Although radio-isotopes are a powerful technique to trace elements such as P, the potential hazards of using them severely restrict their use, especially in the field. Other approaches include measurements of plant height in relation to light penetration through the mixed-species canopy and rooting depth in relation to water depletion [28]. This method suffers, however, due to inter-annual fluctuations in resource levels such as water and nutrients, and general variability in conditions such as soil characteristics and climate between locations [29].

Other studies have used stable isotopes, especially ^15^N tracers to investigate competition for nitrogen (N). For example, localised ^15^N-labelling has been used to investigate the role of root proliferation in interspecific competition for N, but required a contrived system in which competition was restricted to only a small fraction of the plants' root systems [30]. The alternative, more generally used, approach is to simply inject ^15^N sources into soil then measure ^15^N excess in the easily accessible aboveground parts of plants growing on that soil [31]–[34]. However, the usual application of this method takes no account of soil microbial activity which progressively dilutes added ^15^N with unlabelled mineralisation products prior to plant uptake. This dilution effect presents to the plants N sources of constantly varying isotopic signatures, and so obliterates any relationship between the isotopic abundance of the source N pool(s) and that measured in the plant. This means that such studies can effectively estimate the competitive uptake only of added ^15^N tracer, not of the soil N pools themselves. It is the latter that matters ecologically, since competitive success depends not only on the amount of resource captured by a competitor relative to its neighbours, but also on the absolute amount of resource captured as this impinges, via stoichiometry [35] on productivity and, hence, future competitiveness.

However, by combining isotope labelling with models [36], [37] that do account explicitly for the dilution of tracer in the soil pools, ^15^N additions to soil can be used to estimate N (and not just tracer) uptake. Originally developed for use in agricultural settings, these ‘isotope pool-dilution’ approaches have rarely been applied to more complex ecological situations, but are ideal to study plant competition for soil N. It is important to emphasise the clear distinction between pool-dilution methods [36], [37] and simple isotope-labelling experiments [31]–[34]: the former estimate absolute resource (not just isotope) capture over a defined time period; the latter estimate only the relative amounts of isotope (not of the resource itself) captured as fractions of total isotope recovery [38] or of that originally injected [39]. Simple isotope-labelling can, therefore, provide no quantitative information about competitive N (as opposed to ^15^N) capture, a limitation that is rarely appreciated. Simply injecting ^15^N into soil and measuring its subsequent abundance in vegetation without considering the kinetics of microbial N transformations during the labelling period is *not* pool-dilution, and the two approaches should not be confused with one another.

^15^N pool-dilution has additional advantages in that the gross rates of soil N mineralisation are also estimated. These rates reflect the dynamic availabilities of labile N pools (principally NO_3_^−^ and NH_4_^+^, but, potentially, also dissolved organic N [40]). The capture by plants of soil NO_3_^−^ and NH_4_^+^ can therefore be calculated separately even when plants have simultaneous access to those sources. This is another important advantage of isotope pool-dilution over simple tracer experiments, one with particular ecological relevance given the variation among soils in the availability of different N sources, and among plant species in their physiological preferences for alternative sources that are simultaneously available [41] and for which plants can compete.

**Figure 1:**
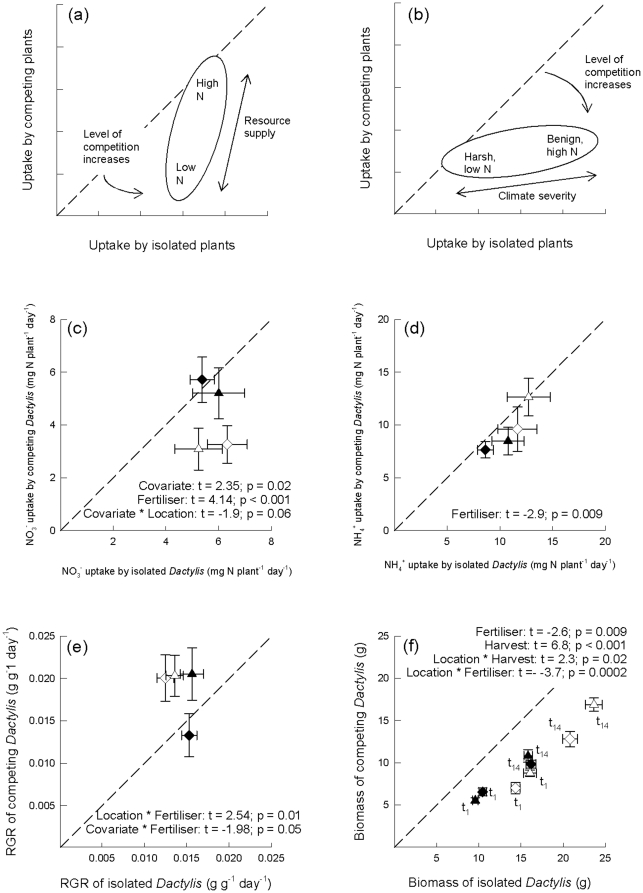
Measures of competition using direct and indirect approaches. (A) Schematic showing results predicted for hypothesis 1 (plant competition for N will increase under conditions of reduced soil nutrient availability); (B) Schematic showing results predicted for hypothesis 2 (plant competition for N will be weaker in a more climatically severe upland environment, and under lower nutrient availability); (C) Uptake of NO_3_^−^; (D) Uptake of NH_4_^+^; (E) RGR over 14 d; (F) Biomass at final harvest. N uptake, biomass and RGR of *Dactylis glomerata* when competing against *Plantago lanceolata* (vertical axes) are plotted against the corresponding measurements for *Dactylis* when growing in isolation (horizontal axes). Symbols indicate either lowland (triangle) or upland (diamond) locations, under conditions of low (shaded) or high N availability (open). Plot f uses the same notation, but in addition, results for the first and second harvests are separately indicated using *t_1_* and *t_14_*, respectively. For plot c, the model explained 40% of the observed variation (4, 31 df); for plot d, 35% (3, 18 df); for plot e, 28% (5,67 df); and for plot f, 62% (6, 150 df). Error bars show 1 standard error of the mean. Dotted lines show the line of equality, i.e. identical uptake, biomass or RGR for competing and isolated *Dactylis*; the further points fall away from the line of unity, the greater the strength of plant interaction (competition below the line, facilitation above) , i.e. the relative difference between uptake, biomass or RGR of isolated and competing plants is greater. Significant results from ANCOVA tests are shown on each plot.

Here, we report an experiment in which we used ^15^N pool-dilution to make *direct* measurements of plant competition for N as an explicit test of alternative theories about variation in the strength and role of competition in relation to environmental conditions. Tilman and others [1], [42]–[44] have argued that the strength of competition remains constant across productivity gradients, but that the key resources for which plants compete shift from being located below-ground under unproductive, nutrient-poor conditions, to above-ground when plants compete for light in productive, nutrient-rich habitats. By inference, this suggests that competition for nutrients is stronger in unproductive habitats and weaker in fertile soils. By contrast, Grime and others [2], [45], [46] argued that competition is less important as an ecological force in more severe environments where plants' ecological success is determined more by genotypic and phenotypic responses to environmental conditions that restrict growth [4] and competition will be stronger under conditions of higher productivity [4]. Despite efforts to conceptually reconcile these alternative theories [47], the lack of a means to measure the process of competition directly and unequivocally has contributed to the enduring impasse. In the study reported here, we measured interspecific competition directly in terms of N capture. We used a classic pot-based experiment with contrasting levels of two types of environmental severity: soil resource supply (low vs. high N availability); and climatic (lowland vs. upland locations). We measured competition directly as the separate, simultaneous uptake of available soil NO_3_^−^ or NH_4_^+^, and indirectly as mean relative growth rate (RGR) over a 14-d ^15^N-labelling period and as final biomass at the end of that period, thus enabling us to compare the direct and indirect estimates. Using this approach with two species common in UK grassland systems, *Dactylis glomerata* L. and *Plantago lanceolata* L., we tested two alternative hypotheses: 1. Interspecific competition increases with reduced soil fertility (see [1]). This will be manifested as smaller uptake of N by competing plants, relative to that by isolated plants, in the low fertiliser conditions compared with the high fertiliser treatments (Fig. 1A); 2. Competition is stronger under conditions of higher soil N and this effect will be the same at both climatically severe and benign environments, although overall uptake of N will be reduced under harsher conditions (see [4]). This will be shown as greater competition for N (a higher negative effect of the impact of neighbours) at the benign lowland site and in the high fertiliser treatment compared to the more climatically severe upland location (Fig. 1B). The novel ^15^N pool-dilution approach we used allowed us to distinguish between these possibilities.

## Results

For NO_3_^−^ (Fig. 1C), there was no difference in uptake by isolated plants due to fertiliser level (mean per capita N uptakes of 5.66±0.5 for low fertiliser and 5.78±0.59 mg g^−1^ d^−1^ for high fertiliser). However, uptake by competing plants in low fertiliser pots was greater (5.43±0.64 mg g^−1^ d^−1^) than those competing at high fertiliser (3.16±0.54 mg g^−1^ d^−1^). There was no difference in uptake between isolated and competing plants (i.e. no competition for NO_3_^−^) in low fertiliser pots, but in the pots with high fertiliser there was strong NO_3_^−^ competition. The ANCOVA test on uptake of NO_3_^−^ showed a strongly significant fertiliser effect (p < 0.001), indicating a highly significant increase in the strength of competition in the high compared to the low N fertiliser treatments. In addition, there was a borderline significant covariate * location effect (p = 0.06). This reflected the fact that, for isolated plants, there was a smaller difference between per capita NO_3_^−^ uptake at the two locations (5.85±0.44 mg g^−1^ d^−1^ at the upland site and 5.59±0.65 mg g^−1^ d^−1^ at the lowland location) than when *Dactylis* competed against *Plantago* (*Dactylis* uptake: 4.62±0.63 mg g^−1^ d^−1^ at the upland site and 4.19±0.66 mg g^−1^ d^−1^ at the lowland site).

For uptake of NH_4_^+^, at both high and low fertiliser, there was no difference in uptake between isolated and competing plants. These points all fell on or very close to the 1∶1 line (Fig. 1D), showing that the presence of *Plantago* did not affect uptake of NH_4_^+^ by *Dactylis* and that no competition for this form of N was occurring. There was a significant fertiliser effect (p = 0.009), but this simply reflected the greater uptake by plants growing under the high fertiliser regime rather than any difference in the strength of competition. This is demonstrated in Figure 1D by the similar increased uptake of both isolated and competing plants under high fertiliser conditions.

Uptake of NH_4_^+^ by *Dactylis* was considerably greater than that of NO_3_^−^: 1.3–4.2 times greater for competing plants, and 1.6–2.4 times greater for isolated plants. This reflected differences in gross rates of ammonification and nitrification, the former being 1.3 to 2.8 times larger than the latter (Table 1). Soil concentrations of NO_3_^−^ were less than 5% of those of NH_4_^+^ (Table 1).

**Table 1:** Concentrations of NH_4_^+^ and NO_3_^−^ in soil and gross rates of nitrification and ammonification.

Location/fertiliser treatment	NH_4_^+^ concentration (µg g^−1^ dry soil)	NO_3_^−^ concentration(µg g^−1^ dry soil)	Nitrification(µg g^−1^ dry soil d^−1^)	Ammonification(µg g^−1^ dry soil d^−1^)
Upland site, high fertiliser	4.492±0.587	0.003±0.000	0.125±0.02	0.158±0.02
Upland site, low fertiliser	1.765±0.202	0.003±0.025	0.088±0.01	0.246±0.01
Lowland site, high fertiliser	1.325±0.232	0.040±0.026	0.095±0.01	0.197±0.01
Lowland site, low fertiliser	0.810±0.468	0.007±0.003	0.128±0.02	0.203±0.01

Values are means ±1 SE. Soil N concentrations were averaged across *t_1_* and *t_14_*. Nitrification and ammonification rates are calculated between *t_1_* and *t_14_* and thus represent rates over the 14 days between harvests.

Over the 14-d period during which competitive N uptake was measured, relative growth rate (RGR) was actually greater for competing than for isolated plants, except for plants in low fertiliser pots at the upland site (Fig. 1E). There was a significant location * fertiliser interaction, reflecting that there was little difference in RGR between competing plants at the two locations under high fertiliser conditions, whereas there was a large difference in RGR between locations under the low fertiliser regime with RGR of competing plants being considerably lower at the upland site. The covariate (isolated *Dactylis*) * fertiliser interaction, was due to the relative difference between RGR of isolated and competing plants. For isolated plants, there was only a small difference in RGR between high and low fertiliser regimes.

In terms of their aboveground biomass, there was no evidence to suggest that plants at the two sites were at different stages of growth. As expected, plants were smaller at the time of the first harvest (*t_1_*), 1 d after labelling, than at the second (*t_14_*), 14 d after labelling. Those receiving high fertiliser were larger than those that received low fertiliser, and isolated plants were bigger than competing plants (Fig. 1F). The significant location * fertiliser interaction reflected the fact that under conditions of low fertiliser there was no difference in biomass due to the location at which plants grew, but under the high fertiliser regime plants at the lowland site had greater biomass than those that grew at the upland site. The location * harvest interaction showed that at the first harvest there was less difference between the biomass of plants at the two locations compared with the second harvest, suggesting that plants growing at the more benign location were able to produce extra biomass at this part of the growing season.

## Discussion

Our first hypothesis was based on Tilman's theory that plant competition is stronger at low levels of fertiliser compared with high fertiliser, and we expected greater competition under low fertiliser conditions [1]. There was a strong effect of N availability on NO_3_^−^ uptake by competing plants (Fig. 1C), but the direction of the effect was opposite to that predicted: stronger competition for NO_3_^−^ occurred in pots with high fertiliser than with low. This evidence contradicts the first hypothesis. It does, however, partly support our second hypothesis that competition is greater at high fertiliser levels [4]. NH_4_^+^ uptake was greater under high fertiliser conditions compared with low (significant fertiliser effect; Fig. 1D), but there was no difference in the strength of competition for NH_4_^+^ between the fertiliser treatments or sites. This experiment appears to confirm the importance of soil fertility as a key driver in plants' competitive interactions, although those interactions were not consistent for the two soil N sources, NO_3_^−^ and NH_4_^+^ (Fig. 1C, 1D). Climatic severity had no impact on strength of competition for either NO_3_^−^ or NH_4_^+^ with only a borderline significant covariate * location effect for NO_3_^−^ uptake. Clearly, when competition is measured directly as simultaneous capture of specific resources, the complexity of the resulting responses is much greater than predicted by existing ecological theories. In the case of competition for soil N, some of this complexity likely arises from the microbial and physico-chemical processes by which N ions are made available in the soil.

Differences in gross mineralisation rates (Table 1) can explain the larger uptake of NH_4_^+^ compared with NO_3_^−^ during this experiment, but there were also large differences in the patterns of NO_3_^−^ and NH_4_^+^ uptake. These were probably caused by a combination of the amounts of each ion available to the plants and their respective mobilities in soil, which in turn were influenced by the experimental treatments. NO_3_^−^ diffuses about ten times faster in soil than NH_4_^+^ [48] and is therefore more easily accessible to plants compared with NH_4_^+^ at any given root length density [49]. The net availabilities of the ions also depend on the rates at which they are produced and consumed, and by their resulting concentrations in the soil solution. Soil NH_4_^+^ concentrations, gross ammonification rates and amounts of NH_4_^+^ taken up during the experiment were greater than for the corresponding NO_3_^−^ figures (Fig. 1C, 1D; Table 1). Therefore, NH_4_^+^ was probably the more plant-available form of N during the experiment. But at the time of measurement, the capture by competing plants of NH_4_^+^ was barely distinguishable from that by isolated plants (Fig. 1D), even though large accumulated differences in biomass production between competing and isolated plants had been established and were associated with both fertiliser supply and location (Fig. 1F). We conclude on the basis of this evidence that the decisive period of competition for NH_4_^+^ had occurred before the time of ^15^N labelling and measurement.

We were able to consider N content (and thus N uptake [36]) in only above-ground biomass. By applying allometric modelling in an experiment with many frequent, destructive harvests, we have shown [50] that when *Dactylis* competes against *Plantago*, its root∶shoot biomass increases considerably during the growing season, whereas that of *Plantago* remains relatively constant. That response can be decisive in determining the superiority of *Dactylis* over *Plantago* over timescales of several weeks as it is associated with greater capture of N and, presumably, other nutrients. But because we were unable to separate roots in this experiment, we cannot evaluate the extent to which that response might have accounted for the effects of species or location on NH_4_^+^ and NO_3_^−^ uptake seen in Figure 1.

As explained in the Introduction, the great (and largely unexploited) advantage of ^15^N pool-dilution techniques in plant competition studies is that they allow the competitive capture of specific soil N pools to be estimated directly, simultaneously and unequivocally. However, they can realistically be applied only over temporal windows 10–20 d long. Characterising the whole competitive process in this way would demand ^15^N-labelling and harvesting successive cohorts of competing and isolated plants. Such experiments would be of truly daunting size, and require a research budget to match. For these reasons, ^15^N pool-dilution approaches are always likely to be limited to certain phases of the competitive process, rather than be applied to an entire competitive trajectory (cf. [50]). The results presented in Figure 1 are therefore quantitative snapshots of the competitive interactions between *Dactylis* and *Plantago* in terms of their NH_4_^+^ and NO_3_^−^ capture, but which, even so, are the first such snapshots to be obtained for any combination of competing plants. We would argue that because the vast majority of plant competition experiments are restricted to aboveground biomass data collected at only one harvest, they, too, provide only snapshots of the interactions between neighbours. Our results have the advantage of directly quantifying competition for N over a defined time-period and in terms of resource capture, not biomass production.

The lack of correspondence between competition for N and final above-ground biomass of plants was unsurprising, given that biomass at the final harvest represents the net accumulation of resources up to the time of harvest and not just the resources (including N) captured over the preceding 14 d. However, the complete absence of any correlation between RGR and N uptake is more surprising, given that these were measured over the same 14-d period. The biomass of isolated plants was greater than that of competing ones (Fig. 1F). But, perhaps surprisingly, competing plants were generally growing more quickly than isolated ones during that period (Fig. 1E). From this experiment we cannot determine the causes of these disparities, but suspect that they are not the result of a genuine facilitative effect. Rather they probably reflect transient growth dynamics, the trajectories of which are masked by the temporal restriction of our study. If so, this highlights the need to consider plants' competitive interactions as dynamic processes. Most plant competition experiments, including those cited by Grime and Tilman in support of their respective theories, are essentially ‘static’ in that their outcomes were measured at only one point in time. This is despite the extensive literature on density-dependent growth and mortality in intraspecific communities that demonstrates that plants' competitive interactions are temporally dynamic [11]. These results presented here clearly highlight the fact that it is possible to come to quite different conclusions about the results of competition experiments, depending on the variable being measured.

### Conclusion

We found no evidence to support Tilman's theory of plant competition, but neither do our results fully support Grime's. These theories do not account for the complexity of the processes that underlie resource supply and capture by competitors. When these processes are measured directly, as in this study, important limitations of the theories are revealed. This is the first study to use an unequivocally direct measure of resource capture to examine the impact of two types of environmental drivers (resource availability and climate) on plant competition, and to compare direct measurements with “proxy” measurements such as biomass and RGR. Although our study was restricted to a limited window of time, we have demonstrated that this powerful technique can be used to study competitive interactions between plants in considerable detail and believe that this technique offers us new insights into these processes. Furthermore, by applying this technique we have shown that in order to further improve our understanding of the environmental regulation of plant competition, theories are required that are based on the reality of resource dynamics, incorporating both temporal variation in the availability and use of resources, as well as differences in their kinetics. These techniques now need field-testing, using mature plants to confirm their validity in more natural systems. In addition, it is important to widen our perspective on plant competition by examining its temporal dynamics (cf. [50]) although isotope pool-dilution will probably not be an appropriate means to do this routinely. We can then begin to understand how the impacts of the environment on the *process* of competition are translated into *outcomes* of competition and ultimately into demographic measures of plant success.

## Materials and Methods

### Plants

We used *Dactylis glomerata* L. a perennial, tussock-forming grass and *Plantago lanceolata* L. a perennial, rosette-forming forb. Both are common, native grassland plants in the British Isles that often grow together [51]. Importantly, they are both competitive, responsive to nutrients and have a similar rooting pattern [52], altitudinal range and phenology [11], [53], [54].

### Locations

The experiment was split between a lowland site (Aberdeen, Scotland, 57°08′N, 2°09′W, elevation 78 m), and an upland site (Braemar, Scotland, 56°59N, 3°29′W, elevation 340 m), the locations are approximately 80 km apart. The upland site represented the practical altitudinal limit for our grassland species as above this altitude the habitat changes to open moorland. Table 2 shows average weather conditions at the two sites between 1960 and 2000 from 1 April to 31 August (the months over which our experiment ran in 2009): although there is little difference in precipitation or average maximum temperatures, the upland site has considerably lower average minimum temperatures. To provide information on environmental conditions specific to the year of the experiment (2009), air (screened) and soil temperatures (5 cm depth) at both sites were recorded for the duration of the experiment (CR800 Data Logger, Campbell Scientific, Loughborough, UK). Precipitation was not recorded at either site as the pots of plants were watered when necessary.

**Table 2:** Weather conditions at the lowland and upland sites, 1960–2000, between 1 April and 31 August each year.

Location	Total precipitation (mm)	Maximum temperature (C)	Minimum temperature (C)
Lowland site	291 (140–507)	14.3 (13.3–15.7)	7.5 (6.8–8.4)
Upland site	296 (125–450)	14.6 (13.3–16.4)	5.18 (4.2–6.3)

Values are means (ranges in parentheses).

### Experimental procedure

**Figure 2:**
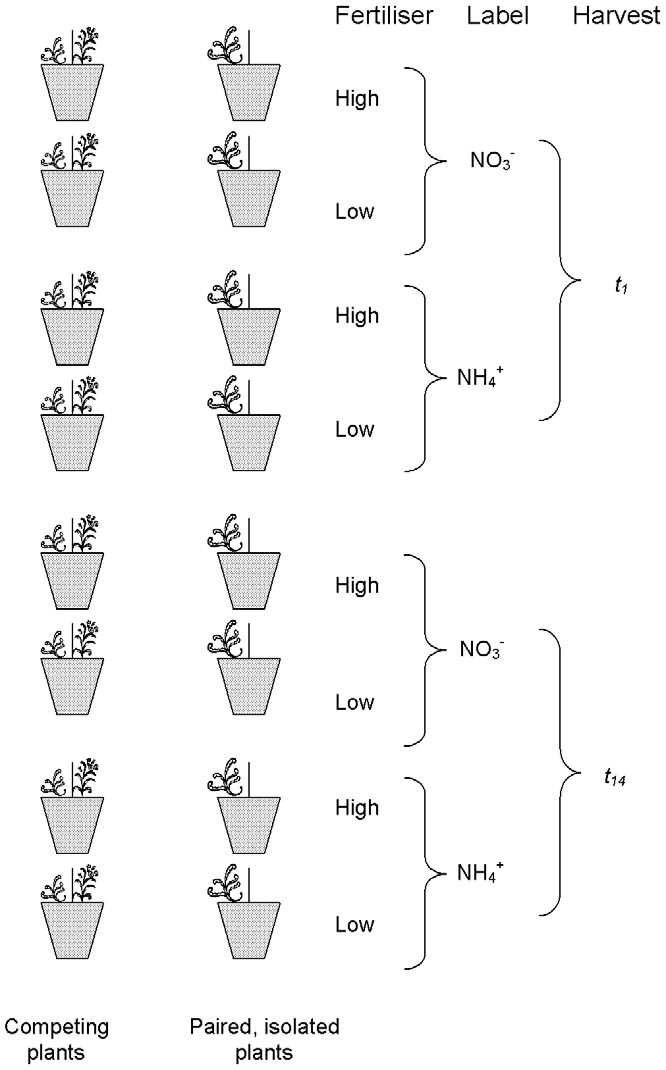
Schematic showing how pots were paired and the different treatments (identical at each location). At each site there were 12 replicates of each treatment combination, arranged with 2 replicates in each block. Pots had a mesh screen which was oriented N-S, with the identity of plant on the east or west of the mesh being randomly selected; similarly with the isolated pots, the plant was randomly assigned to east or west position. Plants received high or low fertiliser, NH_4_^+^ or NO_3_^−^ label and were harvested at *t_1_* or *t_14_*.

Plants were germinated from locally-collected *Dactylis glomerata* and *Plantago lanceolata* seed the previous autumn (2008). Seedlings were over-wintered in an unheated greenhouse so that plants would be of a sufficient size for early transplantation into pots at each site, enabling them to be *in situ* as soon as weather conditions allowed growth. Young plants were transplanted into 15×15×20 cm pots (capacity 3.5 l) and immediately placed at the field sites on 30 March 2009 (lowland site) and 9 April 2009 (upland site). Pots at both locations contained sieved, free-draining, N-deficient sandy loam, from the Countesswells series, pH 6.1. Sufficient P and K was added (30 µg g^−1^ dry soil) to ensure that these were not growth-limiting. Figure 2 shows the treatment combinations. We added NH_4_NO_3_ to half the pots to raise the concentration of extractable inorganic N (NH_4_^+^-N and NO_3_^−^-N) from 3 µg g^−1^ (‘low’ N treatment) to 80 µg g^−1^ dry soil (‘high’ N treatment); determination of soil N concentrations is described below. High fertiliser pots received a further three equal additions of NH_4_NO_3_ totalling 240 µg N g^−1^ dry soil, and low fertiliser pots received a total of 120 µg N g^−1^ dry soil during the experiment. Each pot was planted with either one *Dactylis* plus one *Plantago* growing together, or a single *Dactylis*. Roots were not separated, but mesh screens prevented one plant from over-topping the other so plants should have not been competing for light [14]; screens were oriented N-S and plant identity to the east and west of the screen was assigned randomly, as were the locations of plants in the isolated pot treatments.

Each of 6 replicate blocks at each location (all with identical E-W block orientation) contained two full sets of treatments (plant combination, fertiliser level, ^15^N-label and time of harvest) in a fully factorial design, arranged randomly within each block. There were a total of 192 pots at each site. For pool-dilution calculations [36], each pot was ‘paired’ with an identical pot receiving the same ^15^N label, but which was harvested 14 days after labelling. Within blocks, each of these pairs of competition pots were also paired with a corresponding pair of isolated plant pots for data analysis [55]. Plants at both sites were enclosed in mesh fruit cage to prevent herbivory. Pot sides were covered in silver foil to minimise heat gain by the soil, watered as necessary, and kept weed-free. In order to avoid the effects of time being confounded with other effects of environmental severity, we aimed to harvest plants at a similar developmental point. Weather records for both sites suggested the upland site would be approximately 3 weeks behind the lowland site in terms of air growing degree-days (GDD; a key determinant of plant development) and thus we anticipated that the plants at the two locations – despite having their respective harvests four weeks apart - would be at a similar developmental stage at the time of harvest; thus we calculated GDD to check on the growing conditions that plants at both sites had experienced up to the time of their harvests.

### ^15^N pool-dilution technique

This technique, including its theoretical basis, calculations and assumptions, is described fully elsewhere [36], [37]. It allows the uptake of total N by plants from a ^15^N-labelled soil N pool to be calculated over a defined time period and not, crucially, of only the ^15^N-tracer, as explained in the Introduction. The essential steps are, briefly: (1) measure the concentrations and background ^15^N abundances of plant-available soil N pools (typically NH_4_^+^ and NO_3_^−^); (2) add ^15^N-enriched NH_4_^+^ or NO_3_^−^ to the soil; (3) measure initial concentrations and ^15^N abundances of the soil N pools after 1 d; (4) repeat these measurements after a further 10–20 d; (5) from these, derive gross ammonification and nitrification rates over this period; (6) use these rates to estimate the mean ^15^N excess abundances of the NH_4_^+^ and NO_3_^−^ pools during this period. These are the best practical approximations to the source ^15^N values to which plants have had access, reflecting the progressive ^15^N dilution of the soil pools by unlabelled products of ammonification or nitrification, assuming zero-order kinetics. The estimation of mean pool ^15^N abundances during the labelling period is *the* key feature of the pool-dilution approach and which distinguishes it from simple isotope labelling methods [31]–[34], [39]; (7) use mean pool abundances to derive NH_4_^+^ uptake as *Nx*/*a*, where *N* and *x* are, respectively, the initial N content and atom % excess ^15^N of ^15^NH_4_^+^-labelled plants, and *a* the mean ^15^N excess of soil NH_4_^+^ during the labelling period; (8) calculate NO_3_^−^ uptake similarly using corresponding data from separate ^15^NO_3_^−^-labelled pools and plants. This method requires four sets of pots all receiving the same experimental treatments (in this case, combination of plants, location of pots and fertiliser level; see Fig. 2). Two sets of the pots are labelled with NH_4_^15^NO_3_ and two sets with^15^NH_4_NO_3_. Unlike conventional ^15^N tracer studies, it is necessary to allow an ‘incubation period’ after adding ^15^N to allow thorough mixing of the labelled solution through the soil [37], so the first harvest (*t_1_*) takes place 24 h after labelling, at which time one set of NH_4_^15^NO_3_ –labelled pots and one set of ^15^NH_4_NO_3_ –labelled pots are harvested. This leaves one set of NH_4_^15^NO_3_ –labelled pots and one set of ^15^NH_4_NO_3_ –labelled pots which are harvested 14 d after labelling (*t_14_*) to allow sufficient time to detect changes in plant biomass and total N content. Harvesting 14 d post-labelling is within the timeframe to successfully estimate NO_3_^−^ and NH_4_^+^ uptake by *Lolium perenne* (perennial rye-grass) [36], but longer than that recommended for the estimation of only gross N mineralisation [37]. There is, therefore, some risk of small errors in estimating gross rates due to remineralisation of microbial ^15^N, but these errors would have been spread equally across treatments and would not have biased statistical comparisons. Therefore, a 14-d labelling period was a practical compromise.

After 98 days *in situ*, on 6 July 2009, pots at the lowland site were labelled with 15 mg of labelled NH_4_NO_3_ at a ^15^N enrichment of 99 atom %. The label, in 250 ml of water, was watered onto the soil surface of each pot to ensure uniform distribution throughout the soil, taking care to avoid contacting leaves. Half the pots were harvested 24 h later (*t_1_*), and the remaining pots were harvested 14 d later (*t_14_*), according to the protocol described above. Pots as the upland site were labelled on 3 August 2009, after 116 days *in situ*, four weeks after labelling the lowland pots. This time difference was to allow plants at the two sites to reach approximately similar developmental stages (see above and Fig. 1F). Labelling and harvesting were carried out in the same way at both sites.

### Harvests and sample analysis

The following procedures were undertaken at each harvest. In the lab, root-free soil samples of c. 70 g wet weight (sub-sampled from c. 500 g of soil taken from the pots), were incubated at room temperature for 1 h before extracting NH_4_^+^ and NO_3_^−^ using 2M KCl. After shaking, extractions were filtered and the extract was immediately frozen. NH_4_^+^ and NO_3_^−^ concentrations of soil extracts were measured colorimetrically (Konelab Aqua 20, Thermo, Hemel Hempstead, UK).

Ideally, the intermingled roots of competing plants should be separated and quantified to obtain a full picture of the interaction that has occurred between them, but this is rarely possible in practice [50], [56], which is why almost all plant competition experiments consider only above-ground responses. Root separation is possible with some species' assemblages that happen to have morphologically distinct roots [39], or by using differences in ^13^C natural abundance if the competitors are a combination of C_3_ and C_4_ species [57], [58], but neither is the case with *Dactylis* and *Plantago*. Consequently, biomass and N/^15^N contents of only above-ground parts of the competing plants could be estimated reliably in this experiment. Above-ground biomass (mainly leaves) of each plant was separated from roots at the soil surface, oven-dried (80 C) to constant weight and weighed. Total N and ^15^N contents of harvested biomass samples were determined by isotope ratio mass spectrometry (ANCA-NT isotope ratio mass spectrometer with ANCA-NT Solid/Liquid Preparation Module; Europa Scientific Ltd, Crewe, UK). At the first harvest, roots from 10 randomly-selected pots were treated and stained [59] for determination of arbuscular mycorrhizal (AM) colonisation [60] because plants' competitive interactions can be influenced by AM fungi [61]. No colonisation was found and the remainder of the plants were assumed to be AM-free.

Soil NH_4_^+^ and NO_3_^−^ were prepared for isotopic analysis [62]. This is a two-part process in which the NH_4_^+^ and NO_3_^−^ moieties are serially converted into NH_3_ using different reagents so that N extracted from soil is in a form that can be isotopically analysed. First ^15^NH_3_ is evolved from ^15^NH_4_NO_3_ and trapped, then the same soil extract is treated to evolve ^15^NH_3_ from NH_4_^15^NO_3_ which is again trapped. Analyses of sub-samples of each extract revealed very low N concentrations (<1.5 µg l^−1^), so to ensure sufficient N for detection by mass spectrometry, 40 µg N as an unlabelled NH_4_NO_3_ solution was added to each sample. Extracts were sealed in gas-tight jars following addition of 0.7 g MgO and two Whatman No 1 filter paper discs (5 mm diameter), each acidified with 5 µl 2.5M KHSO_4_ suspended from the lid of the jars to trap NH_3_ evolved from the solutions. After one week, the jars were opened and the discs removed and dried in a desiccator, then analysed for ^15^N. Diffusions from soils labelled with ^15^NH_4_NO_3_ were then completed. For soils labelled with NH_4_^15^NO_3_, and thus requiring the second diffusion step, two new acidified discs were placed in the lid of these jars which were then resealed, following addition of 0.2 g MgO and 0.4 g Devarda's alloy and then treated as described above.

### Calculations and statistical analyses

The pool-dilution method (see above) requires pots of plants to be paired to provide data for calculations for each of the two harvests, *t_1_* and *t_14_*. These paired plants from the destructive harvests were also used to calculate mean aboveground RGR (between *t_1_* and *t_14_*, for direct comparison between N uptake and RGR over the same time period) using the standard method [55]:
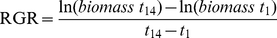
(1)
*Dactylis* does not grow when temperatures are below 5.6°C [53] so growing degree days (GDD) were calculated as:
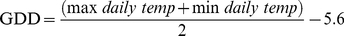
(2)Cumulative GDD up to the harvests at *t_14_* was calculated by adding GDD for each day, when GDD>0. Cumulative GDD for air temperatures was 837 at the lowland site and 667 at the upland; and for soil temperatures it was 845 and 996 respectively, perhaps indicating differences in radiant heat and air temperature between the two sites.

^15^N enrichments of the acidified discs were corrected to account for the additional NH_4_NO_3_ added using a mixing equation [63]. ^15^N-pool dilution calculations followed procedure A of Barraclough [36]: ^15^N-pool dilution calculations required that the above-ground biomass at the second harvest was greater than that at the first. Given the complexity of the experimental design and to prevent any bias, pots had to be paired at the start of the experiment and any alteration of this at harvest to take into account differences in plant sizes would have unbalanced the other treatments. Although plants were generally larger at the second harvest, variability between paired pots meant that this was not always the case and resulted in the loss of some data. In addition, 24 NH_4_^+^ and 1 NO_3_^−^ samples were also lost during analysis. Pairing the dependent variable with its covariate by block in the data analysis resulted in further loss of data if either covariate or dependent variable had been lost. This resulted in sample sizes of 36 (out of a possible 48 data points) for uptake of NO_3_^−^, 22 (out of a possible 48 data points) for NH_4_^+^ uptake, 73 (out of a possible 96 data points) for RGR and 159 (out of a possible 192) for biomass. For all tests, residuals were tested for normality and heteroscedasticity and transformed where required. Block was tested separately against each dependent variable but showed no significant effects (P>0.05).

We analysed the data using uptake of NO_3_^−^ and NH_4_^+^, RGR and above-ground biomass of *Dactylis* plants both in competition and in isolation. To test the competitive effect of *Plantago* on *Dactylis* (note that this is the same as the competitive response of *Dactylis* to *Plantago*) at different locations and fertiliser levels, we ran separate ANCOVA tests for uptake of each ion, RGR and biomass using *Dactylis* when growing with a neighbouring *Plantago* as the response variable, and *Dactylis* growing alone (paired from the same block) as the covariate; location and fertiliser served as fixed effects. Using ANCOVA in this way allowed us to test for the effects of competition under the different treatments, whilst taking into account any differences in N uptake, biomass or RGR due to those different treatments [24] but avoiding the use of statistically problematic competition indices [22]. When analysing above-ground biomass, harvest date (i.e. *t_1_* or *t_14_*) was also included as a fixed effect.

These tests were run as linear models in R [64] and simplified by comparing the explanatory power of models from which non-significant interaction terms had been removed [65]. Models included all possible two-way interactions. Significant covariate * treatment interactions indicate that the slopes of the regression lines are not homogeneous. Whilst this is generally considered to be a violation of the assumptions of ANCOVA, such interactions show that the treatments affect the relationship between the dependent variable and its covariate and these effects can be of great interest [66].

In relation to this study, significant main effects need not necessarily reflect differences in competition but significant interactions are of greater interest: fertiliser * location interactions show that plants do not respond to the addition of fertiliser in the same way at both locations; a covariate * location interaction shows that, given a change in the response variable in the covariate (isolated plant) the competing plants do not respond in the same way at the two sites. Similarly, a significant covariate * fertiliser interaction shows that the covariate (isolated plant) has responded differently from the competing plants to the fertiliser treatment. Where there are significant covariate interaction terms, it is difficult to interpret main effects as the interpretation of these will change according to the value of the covariate [66] so, where these are present, we concentrate on the interaction terms rather than significant main effects. Results are presented using treatment contrasts to overcome issues of ordering variables within each model.

The data were plotted (Fig. 1) to show NO_3_^−^ and NH_4_^+^ capture by *Dactylis* when growing with a neighbour (vertical axis) and when growing in isolation (horizontal axis). This shows clearly the effect of a neighbouring plant: where data lie along the 1∶1 line, there is no difference in performance (however measured) between plants with a neighbour and those growing in isolation, demonstrating that there is no effect of competition. When data are below the 1∶1 line, plants growing with a neighbour perform worse than isolated plants, showing that competition is occurring. Conversely, if data fall above the 1∶1 line, competing plants out-perform their isolated counterparts (i.e., facilitation, not competition, is occurring [67]). The further the points fall below the 1∶1 line, the greater the effect of a competitor (i.e., the relative difference between competing and isolated plants becomes larger); these differences are illustrated by the schematics in Figure 1A, 1B. As noted above, using statistical analyses alone to interpret these data could be misleading as, for example, a significant fertiliser effect need not necessarily be due to differences in competition, simply that different amounts of N were taken up, in which case all the points would fall along the 1∶1 line. Similarly, it is necessary to check that significant interaction terms in the model relate to the occurrence of competition.

The schematic Figure 1A and 1B show the results we expected depending on whether hypotheses 1 or 2, respectively, was correct. Hypothesis 1 is based on Tilman's theory that competition is stronger when resources are scarce (e.g., under low fertiliser conditions). Hence, in Figure 1A we expected the data for N uptake under high fertiliser conditions to lie at the top of the oval and uptake under low fertiliser at the bottom end. We did not anticipate different responses from plants growing at the different locations, as resource supply is seen as the primary factor controlling competition. We did, however, expect greater uptake by all plants (regardless of competitive effects) under the high fertiliser regime, and so the oval is slightly tilted with respect to the 1∶1 line. Hypothesis 2 is based on the expectation from Grime's model that competition is less important relative to other factors under harsher conditions. In Figure 1B, the ‘benign’ end of the oval is tilted further away from the 1∶1 line. Although we expected all plants to take up absolutely more N under better growing conditions at the benign site, competition is also expected to be stronger here, and so there is a greater deviation away from the 1∶1 line. In addition, we expected competition to be stronger under the high fertiliser compared with the low fertiliser treatments.
